# Neuroimmunology of Huntington's Disease: Revisiting Evidence from Human Studies

**DOI:** 10.1155/2016/8653132

**Published:** 2016-08-08

**Authors:** Natalia P. Rocha, Fabiola M. Ribeiro, Erin Furr-Stimming, Antonio L. Teixeira

**Affiliations:** ^1^Neuropsychiatry Program, Department of Psychiatry and Behavioral Sciences, McGovern Medical School, The University of Texas Health Science Center at Houston, Houston, TX 77054, USA; ^2^Department of Biochemistry and Immunology, ICB, Universidade Federal de Minas Gerais, 31270-901 Belo Horizonte, MG, Brazil; ^3^Department of Neurology, McGovern Medical School, The University of Texas Health Science Center at Houston, Houston, TX 77030, USA

## Abstract

Huntington's disease (HD) is a neurodegenerative disorder characterized by selective loss of neurons in the striatum and cortex, which leads to progressive motor dysfunction, cognitive decline, and psychiatric disorders. Although the cause of HD is well described—HD is a genetic disorder caused by a trinucleotide (CAG) repeat expansion in the gene encoding for huntingtin (*HTT*) on chromosome 4p16.3—the ultimate cause of neuronal death is still uncertain. Apart from impairment in systems for handling abnormal proteins, other metabolic pathways and mechanisms might contribute to neurodegeneration and progression of HD. Among these, inflammation seems to play a role in HD pathogenesis. The current review summarizes the available evidence about immune and/or inflammatory changes in HD. HD is associated with increased inflammatory mediators in both the central nervous system and periphery. Accordingly, there have been some attempts to slow HD progression targeting the immune system.

## 1. Introduction

Huntington's disease (HD) is a neurodegenerative disorder characterized by selective loss of neurons in the striatum and cortex, which leads to progressive motor dysfunction, cognitive decline, and psychiatric disorders [1]. HD is a genetic disorder caused by a trinucleotide (CAG) repeat expansion in the gene encoding for huntingtin (*HTT*) on chromosome 4p16.3. The normal* HTT* presents less than 36 CAG repeats. Mutated* HTT* with 40 or more CAG repeats results in HD with complete penetrance. Expansions varying from 36 to 39 CAG repeats may also result in HD, but with incomplete penetrance [2, 3]. The CAG repeat expansion in* HTT* encodes an expanded polyglutamine tract in the huntingtin (HTT) protein resulting in a mutant protein [1].

The physiological functions of HTT are still a matter of debate. A range of evidence points to HTT's central role in embryonic development. HTT has also been implicated in controlling brain neurotrophic factor (BDNF) production, neuronal gene transcription, and synaptic transmission [4]. The neuronal death observed in HD might result from loss of function and/or gain of toxicity of mutant HTT, but a range of evidence suggests that HD arises mainly from gain of toxicity from an abnormal conformation of mutant HTT [5]. Although the cause of HD is well known and the mutant HTT is pivotal to HD pathophysiology, the ultimate cause of neuronal death is still uncertain. Apart from impairment in systems for handling abnormal proteins, other metabolic pathways and mechanisms might contribute to neurodegeneration and progression of HD [1]. Among these, inflammation seems to play a role in HD pathogenesis (reviewed in [6]).

At first, neuroinflammation can be regarded as beneficial for neuronal tissue since it promotes clearance of cell debris, but scenario is definitely more complex. For instance, apart from modulating immune cell functions, inflammatory mediators also act on neurons, possibly contributing to neuronal death. Neuronal death further activates inflammatory mechanisms, resulting in a vicious cycle of inflammation and neurodegeneration [7]. Besides inflammatory processes occurring in the central nervous system (CNS), systemic inflammation might contribute to the neuronal damage in HD [8].

The objective of the current review is to summarize the available evidence about inflammatory/immune changes in HD. Herein, we showed that HD is associated with increased inflammatory mediators in both CNS and periphery. In addition, we briefly presented the studies targeting immune disruption as an attempt to slow disease progression.

## 2. Central Nervous System Inflammation

### 2.1. Postmortem Studies

Robust evidence regarding neuroinflammation in HD comes from postmortem studies with brain tissue from patients with HD. The term neuroinflammation was initially used to describe the infiltration of peripheral immune cells in the CNS in response to infectious agents or trauma [6]. This term is currently also used to describe the inflammatory process associated with neurodegeneration.

The pathological hallmark of HD is prominent degeneration of the caudate and putamen (collectively known as striatum), along with accumulation of nuclear and cytoplasmic inclusions of mutant huntingtin in neurons. Postmortem studies also showed significant microglial activation in brain regions implicated in HD pathogenesis, that is, striatum, globus pallidus, and cortex [9, 10]. Interestingly, the number of activated microglia in the striatum and cortex correlated with neuronal loss, corroborating the view that neuroinflammatory changes may be elicited by degenerating neurons [10]. Indeed, infiltration of peripheral inflammatory cells is not usually seen in HD [9–11]. These results regarding microglial activation in postmortem human brains can be recapitulated in mouse models of HD. It has been shown that mutant HTT was expressed in murine microglial cells, facilitating autonomous microglial activation, which was sufficient to trigger neurodegeneration [12]. As observed in patients, increased microglial activation was detected in the striatum of presymptomatic R6/2 mice (mouse model expressing human mutant HTT exon 1 containing 150 CAG repeats) [13]. In addition, macrophages and microglia derived from R6/2 and YAC128 (expressing the full-length human HTT gene containing 128 CAG repeats) were also more active in response to stimulation [8, 14]. Notably, changes in striatal and microglial morphology were shown to be age-dependent in YAC128 mice [15].

In parallel with microglial activation, increased number of astrocytes has also been described in postmortem brains of patients with HD [9, 11]. The onset of microglial activation and astrocytosis might differ depending on the neuropathological severity grade (based on the severity of atrophy and neuronal loss). No reactive astrocytes were observed in grade 0 (no abnormality on conventional neuropathological evaluation) with increase in other stages. In contrast, the increase in the density of microglial cells was already observed in grade 0, with modest increase from grade 0 to 3 and significant increase in grade 4 over controls [9]. While the increase in microglial activation seemed to be a very early event, being present even before neuropathological changes, reactive astrocytosis was observed only after neurodegeneration. Both microglial activation and reactive astrocytosis grades correlate with disease severity.

Microglial activation and astrocytosis result in increased production of inflammatory mediators. Interleukin- (IL-) 6, IL-8, tumor necrosis factor- (TNF-) *α*, monocyte chemoattractant protein- (MCP-) 1/CCL2, and IL-10 mRNA levels were markedly increased in the striatum of patients with HD in comparison with controls [8, 16]. In addition, upregulated expression of IL-6, IL-8, and matrix metalloproteinase- (MMP-) 9 was also described in the cortex and cerebellum of patients with HD [16].

Given the important role of astrocytes in supporting neurons, studies have speculated whether astrocytic dysfunction might be involved in neurodegeneration in HD. Marked accumulation of the chemokine regulated on activation normal T cell expressed and secreted (RANTES/CCL5) was found in astrocytes in the frontal cortex, substantia nigra, and caudate of patients with HD, but not in astrocytes of matched controls. No difference in the cytosolic MCP-1/CCL2 was found in astrocytes from patients and controls [17]. Moreover, the expression of the amino-terminal mutant HTT (160Q) exclusively in astrocytes was sufficient to induce neurological symptoms in mice [18].

Intracellular signaling pathways regulate the expression of genes influencing a broad range of biological processes, including innate and adaptive immunity, inflammation, and stress responses. The nuclear factor kappa B (NF-*κ*B) is a major downstream transcription factor that is responsible for promoting the transcription of inflammatory mediators upon stimulus. Abnormal activation of the NF-*κ*B pathway occurred in astrocytes of patients with HD. The nuclear localization of p65 (a subunit of NF-*κ*B) was found in GFAP-positive astrocytes in the caudate nucleus of patients while no nuclear localization of p65 was observed in matched controls [19]. NF-*κ*B signaling cascade acts in parallel with other pathways, including the signaling pathways initiated by phosphatidylinositol 3-kinase (PI3K) and protein kinase B (PKB, also known as Akt). Changes in Akt levels were also described in the striatum of patients with HD. Caspase-3-generated Akt product (Akt 49 kDa) was found in cerebellum and cortex from patients, but not from controls [20]. The same work group showed later that Akt is decreased by caspase-3 cleavage in the brains of patients with HD [21].

### 2.2. Positron Emission Tomography (PET) Studies

An interest in evaluating microglial activation* in vivo* has been raised mainly due to the results obtained from postmortem studies. A growing body of evidence has shown that microglial activation as assessed* in vivo* using positron emission tomography (PET) is associated with HD progression.

^11^C-PK11195 PET is an* in vivo* marker of activated microglia. The peripheral benzodiazepine binding sites for the isoquinoline ^11^C-PK11195 are expressed by the mitochondrial membranes of activated but not resting microglia/brain macrophages. ^11^C-raclopride PET is a marker of dopamine D2 receptor binding and hence striatal GABAergic cell function. A significant increase in striatal ^11^C-PK11195 binding was found in patients with HD compared with controls. ^11^C-PK11195 binding significantly correlated with disease severity as assessed by striatal reduction in ^11^C-raclopride binding, the Unified Huntington's Disease Rating Scale (UHDRS) score, and the number of patients' CAG repetitions. Increase in microglia activation in cortical regions of patients was also noticed [22].

Presymptomatic HD gene carriers also presented increased striatal ^11^C-PK11195 binding which was associated with the severity of striatal neuronal dysfunction evaluated by ^11^C-raclopride PET [23]. These data were corroborated by a later study that combined magnetic resonance imaging (MRI) and PET analyses. Worsening in atrophy evaluated by MRI was accompanied by a reduction in ^11^C-raclopride and an increase in ^11^C-PK11195 bindings in patients with HD. In premanifest HD, increased level of microglial activation in the associative striatum and in the regional network associated with cognition correlated with 5-year probability of HD onset [24].

Based on the alterations in metabolism, sleep, and circadian rhythms as well as in the hypothalamic-pituitary axis described in HD, one study aimed to evaluate* in vivo* D2 receptor's loss/dysfunction and changes in microglial activation in the hypothalamus of patients. A significant decrease in mean hypothalamic ^11^C-raclopride binding and a significant increase in ^11^C-PK11195 binding were reported in patients with HD in comparison with controls. These pathological changes occur very early in the course of the disease since they were observed in both presymptomatic and symptomatic patients [25].

More recently, increased microglial activation in somatosensory cortex (as evaluated through ^11^C-PK11195 binding) was associated with higher plasma levels of IL-1*β*, IL-6, IL-8, and TNF-*α* in premanifest HD gene carriers [26].

### 2.3. Cerebrospinal Fluid Analysis

Another way to investigate inflammatory changes in the CNS in living patients is using cerebrospinal fluid (CSF) samples. The complement factors C1QC, C2, and C3 and other proteins associated with inflammatory pathways, namely, peptidoglycan recognition protein 2 (PGLYRP2) and apolipoprotein A4 (APOA4), were found to be increased in CSF from patients with HD in comparison with controls. CSF levels of these proteins followed the trend consistent with elevating as disease progressed (control < HD-early stage < HD-mid stage) [27]. Clusterin was also increased in the CSF of patients in comparison with controls [28]. Clusterin is a molecular chaperone associated with the clearance of cellular debris and apoptosis and is intimately associated with the regulation of complement-mediated membrane attack [29]. It is possible that the elevated level of these complement related proteins could contribute to disease progression. However, R6/2 mice crossed with complement C3 deficient mice exhibited no alteration in multiple behavioral assays, weight, and survival [30].

Glia cells produce matrix metalloproteinases (MMPs) and free radicals in response to proinflammatory stimuli. -MMPs and their regulators, tissue inhibitors of metalloproteinases (TIMPs), play important roles in inflammation and wound healing. MMP-3 and MMP-9 levels are increased in CSF from patients with HD in comparison with controls [31]. Moreover, MMP-3, MMP-9, and TIMP-1 levels were associated with disease severity in HD as assessed by motor scores. MMP-3 is an endogenous neuronal activator of microglia and also induced increased cytokine release by microglia in YAC128 mice, that is, mice expressing full-length mutant human huntingtin with 128 polyglutamine repeats [31].

However, not all studies showed altered levels of inflammatory/immune markers in the CSF of patients with HD. For instance, there was no difference in CSF levels of YKL-40 between patients and controls [32]. YKL-40 (CHI3L1) is a protein usually upregulated in inflamed tissues in several inflammatory diseases. Astrocytes and microglia cells express YKL-40 in the brain, and its level is significantly elevated in different acute and chronic neuroinflammatory diseases. The meaning of this study may be twofold: CSF level of YKL-40 is not suitable as HD marker, and YKL-40 may be altered only in overt inflammatory conditions.

In order to investigate the relationship between central and peripheral inflammatory processes, IL-6 and IL-8 levels were measured in matched plasma and CSF samples from patients with HD and controls. CSF and plasma levels of IL-6 and IL-8 correlated closely in both HD and controls' samples. Unfortunately, authors did not compare CSF levels of these molecules between samples from HD and controls subjects [8].

## 3. Peripheral Inflammation

The evidence of microglial activation and changes in CSF inflammatory-related proteins levels pointed to immune changes in HD. Given the CNS and immune system cross-talk, it is reasonable to hypothesize that patients with HD present changes in peripheral inflammatory/immune parameters. The search for changes in the peripheral immune system is important for helping not only to elucidate HD pathophysiology but also to identify biomarkers useful for noninvasive monitoring of disease progression and/or response to treatment.

### 3.1. Peripheral Cells Dysfunction

Nearly one decade before the discovery of the gene that causes HD, one study had speculated that gene defects could be expressed in nonneural as well as neural cells. Attempting to prove this hypothesis, Gollin et al. evaluated abnormalities in peripheral blood lymphocytes. Using flow cytometry combined to the fluorescent membrane probe 8-anilino-l-naphthalene sulfonate (ANS), they observed an increase in ANS fluorescence intensity in lymphocytes harvested from patients with HD in comparison with controls [33]. Decades later, studies confirmed this hypothesis, showing that mutant HTT can be found in several cells types, including lymphocytes [6].

Although HTT is an ubiquitously expressed constitutive protein,* HTT* mRNA expression is higher in immune cells in comparison with other cell types [6, 34]. Mutant HTT was detected in monocytes, T cells, and B cells of patients with HD. In addition, mean mutant HTT levels increased steadily with disease progression, with significant differences between premanifest patients (HD gene carriers) and patients with HD (manifest HD patients). Monocyte and T cell mutant HTT levels were significantly associated with disease burden scores and caudate atrophy rates in patients with HD [35].

Abnormal levels of mutant HTT in peripheral immune cells might result in changes in these cells functions. Mutant HTT appears to produce functional overactivity of monocytes. For instance, monocytes from HD mutation carriers (premanifest subjects) stimulated with lipopolysaccharide (LPS) displayed excess IL-6 production compared with cells from control subjects [8]. In addition to IL-6, monocytes from patients with HD produced higher levels of TNF-*α* and IL-1*β* after LPS stimulation when compared with controls. Also macrophages from patients produced higher levels of TNF-*α* and IL-8 than controls [36]. These data show that myeloid cells isolated from patients with HD are hyperreactive, producing elevated levels of several key proinflammatory cytokines following stimulation. Interestingly, lowering HTT expression using small interfering RNA reversed HD myeloid cell hyperreactivity [36]. This finding corroborates the view that abnormal levels of mutant HTT impact on monocyte function.

Lymphocyte dysfunction has also been described in HD. Peripheral lymphocytes harvested from patients with HD produced a “migration inhibitor factor” when exposed to brain tissue from HD. The same did not occur when lymphocytes from control subjects were exposed to the same tissue. HD lymphocytes also responded to presence of multiple sclerosis brain tissue with a “migration inhibition factor” production [37]. Further* in vitro* and* in vivo* evidence show that mutant HTT impairs immune cell migration. Using well-characterized mouse models of HD (i.e., YAC128 and BACHD mice that express full-length HTT and present many behavioral and pathological features of the disease), Kwan et al. (2012) found that primary microglia from early postnatal HD mice were profoundly impaired in their migration to chemotactic stimuli. The expression of a mutant HTT fragment in microglial cell lines was sufficient to reproduce the impairment in migration. Microglia expressing mutant HTT presented a retarded response to brain injury* in vivo*. Leukocyte recruitment was impaired after induction of an inflammatory stimulus (i.e., peritonitis) in HD mice. This impairment was normalized upon genetic deletion of mutant HTT in immune cells. In line with these experimental results, migration of monocytes harvested from premanifest HD subjects upon chemotactic stimuli was also severely impaired [34].

Recently, Miller et al. (2015) evaluated whether patients with HD and controls exhibit differences regarding the frequency of blood lymphocytes. The frequency of circulating T lymphocyte subsets did not differ between HD and controls. The activation levels of CD4+ and CD8+ T lymphocytes were also assessed by CD62L^low^ expression. Again, no significant differences were seen between the HD and control leukocytes populations [38]. The ability of lymphocytes to rapidly proliferate in response to a stimulus is a key feature of the adaptive immune system. The proliferative response of T lymphocyte populations (CD3+, CD3+CD4+, and CD3+CD8+) was not impaired in HD in comparison with controls in response to stimuli (either anti-CD3 and anti-CD28 antibodies or PHA-P). CD4+ lymphocytes from patients with HD and controls did not differ regarding the production of cytokines [IL-2, IL4, IL-6, IL-8, IL-5, IL-13, interferon- (IFN-) *γ*, IL-10, IL-1b, IL-12p70, and TNF-*α*] upon anti-CD3/CD-28 antibodies stimulation [38]. The authors concluded that peripheral immune dysfunction in HD is likely to be mediated primarily by the innate rather than the adaptive immune system. However, the conclusions based on this work are limited due to the small sample size (varying from 5 to 12 subjects per group). Studies comprising larger samples are needed in this regard.

Corroborating the hypothesis of normal adaptive immune response in HD, plasma levels of immunoglobulin G (IgG), IgA, and IgM were found to be unchanged in patients [8]. Conversely, one study found that patients with HD presented higher serum levels of IgA than controls. In addition, patients who died within one year after laboratory measurement presented higher levels of IgM, circulating immune complexes and neopterin compared to survivors [39].

### 3.2. Circulating Levels of Inflammation-Related Molecules

Peripheral immune cell infiltration is not observed in the brain of patients with HD. Nevertheless, as there is an intimate interaction between peripheral and CNS immune system, changes in one of them may influence the other and vice versa. Based on this assumption, abnormalities in circulating (i.e., blood) levels of inflammatory molecules were also investigated in HD. Inflammatory changes can occur in early stages of the disease, even in preclinical stages. For instance, C-reactive protein (CRP) levels were increased in premanifest HD subjects compared to controls [40]. The majority of studies found that inflammatory molecules levels increase as the disease advances. Plasma levels of IL-6 were increased in moderate HD in comparison with both controls and early HD patients [28]. Not only IL-6, but also IL-8 levels increased across groups from controls to different stages of the disease. IL-6 levels were increased in HD gene carriers with a mean of 16 years before the predicted onset of clinical symptoms. TNF-*α*, IL-10, and IL-4 were increased only in moderate stage HD patients in comparison with controls [8]. In addition, plasma levels of IL-8 and TNF-*α* correlated with clinical symptoms, as evaluated by the UHDRS. Cytokines involved in the innate immune response (IL-6 and IL-8) were increased in the earlier stages of disease (even 16 years before the predicted onset of clinical symptoms), while anti-inflammatory cytokines IL-10 and IL-4 increased only in moderate stage of disease; therefore, the authors proposed that early innate immune activation could be a target in the development of disease-modifying therapies [8]. Further evidence of immune changes tracking with disease progression came from chemokines assessment. Plasma levels of the chemokines CCL26/eotaxin-3, CCL4/macrophage inflammatory protein- (MIP-) 1*β*, CCL11/eotaxin, CCL2/MCP-1, and CCL13/MCP-4 were statistically elevated above control levels in HD, with the first three increasing linearly with disease progression [41].

Agreeing with these results, one study described that plasma levels of proteins involved in regulation of the innate immune system, namely, alpha-2-macroglobulin (A2M), C7, C9, and clusterin, are increased in patients with HD in comparison with controls. The levels of these proteins were associated with disease progression. Plasma clusterin levels raised in parallel with CSF levels in HD [28].

Data obtained in mouse models were similar to those seen in patients with HD, as plasma levels of several inflammatory molecules, including IL-6, IL-8, TNF-*α*, IL-10, IL-1*β*, and IL-12p70, were elevated in the HD mouse models R6/2, HdhQ150 (murine model expressing 150 CAG repeats inserted in the mouse endogenous HTT gene first exon), and YAC128 [8, 12, 42]. IL-6 is particularly important in HD as the administration of an antibody that neutralizes IL-6 into R6/2 mice decreased weight loss at late stages and partially rescued motor deficits [43].

Some studies failed to show changes in the circulating levels of inflammatory molecules. For instance, the levels of adipokines, namely, leptin and adiponectin, were not changed in HD [40]. Accordingly, plasma levels and diurnal rhythmicity of leptin, adiponectin, and resistin were not significantly different between patients and controls. However, when corrected for fat mass, mean plasma leptin concentration as well as basal, pulsatile, and total secretion rates increased with the size of the CAG repeat mutation. Both higher pulsatile leptin secretion and higher mean adiponectin levels were associated with a greater degree of motor and functional impairment in patients with HD. CAG repeat size-dependent interference of the HD mutation with adipose tissue function may contribute to weight loss in patients with HD [44].

Wang et al. (2014) did not find any difference in circulating levels of TNF-*α* between patients with HD and controls [40]. It is worth mentioning that TNF-*α* does not seem to be a stable molecule, and its soluble receptors, which are released in response to changing levels of TNF-*α*, seem to be more reliable markers of TNF-*α* activity than TNF-*α* itself [45]. In this sense, serum levels of soluble TNF receptor type I (sTNFRI) were increased in patients with HD [39].

In sum, we found that patients with HD exhibit increased circulating levels of inflammatory molecules, mainly related to innate immunity. This increase is already observed in early stages of the disease, that is, years before the onset of motor symptoms. It remains to be further investigated whether inflammatory/immune mediators can be used as biomarkers for disease progression and treatment response. This is a special need raised by clinical studies investigating the efficacy of disease-modifying drugs in HD.

### 3.3. Intracellular Signaling Cascades

Intracellular signaling pathways have been investigated in peripheral blood cells in HD. The most commonly activated signaling pathway downstream of cytokine receptors is the janus kinase/signal transducer and activator of transcription (JAK/STAT) pathway, coordinating cytokine-mediated gene expression and repression. Using flow cytometry, Träger et al. (2013) investigated the JAK/STAT pathway in monocytes from patients with HD. At baseline, while both phosphorylated (p)STAT1 and pSTAT3 levels were unchanged, pSTAT5 levels were significantly elevated in HD gene carriers' monocytes compared with control cells. Targeted activation of JAK/STAT signaling molecules using specific STAT1, STAT3, and STAT5 activators (IFN-*γ*, IL-6, and granulocyte-macrophage colony-stimulating factor (GM-CSF), resp.) found the same level of pathway activation in control and HD monocytes, indicating normal function of the signaling cascade in HD [46].

It has been demonstrated that the I*κ*B kinase/NF-*κ*B signaling pathway, which promotes cytokines and chemokines release, was upregulated by mutant HTT contributing to neurotoxicity [47]. HTT bound I kappa B kinase (IKK) complex in a CAG repeat length dependent manner [36]. Activation of the IKK complex leads to the phosphorylation and degradation of I*κ*B, the endogenous inhibitor of NF*κ*B. I*κ*B was degraded more rapidly and over a prolonged period of time in monocytes from patients with HD when compared to controls, as a result of IKK activation. In addition, levels of phosphorylated I*κ*B were increased in monocytes isolated from patients with HD compared with control subjects [36].

One study demonstrated a progressive but marked alteration of a prosurvival pathway in HD and further implicated it as a key transduction pathway regulating the toxicity of HTT. Levels of Akt levels were twice higher in immortalized lymphoblasts from patients with HD than controls, but the amount of activated Akt (pAkt S473) was not different. Accordingly, the active Akt/total Akt ratio in patients was significantly lower than that in controls. The same pattern of Akt and pAkt observed in immortalized lymphoblasts was found in primary lymphocytes [21].

**Figure 1:**
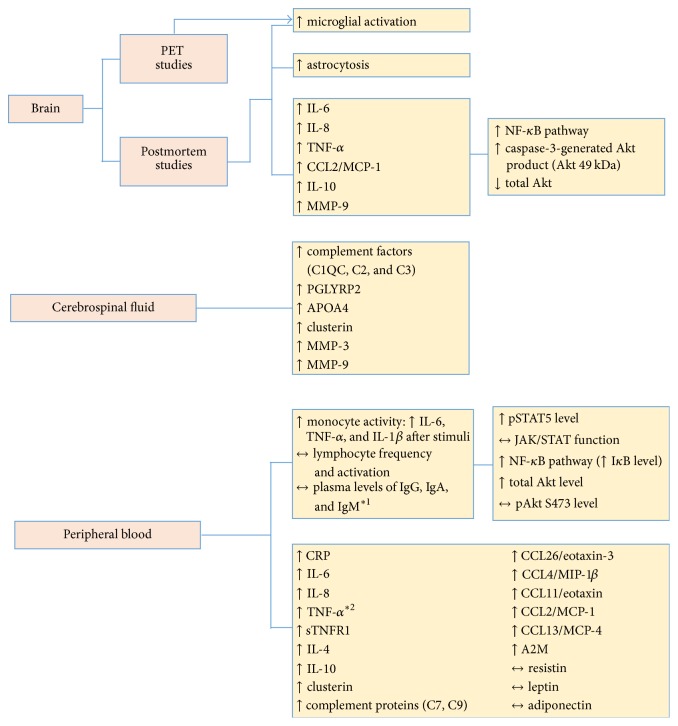
Inflammatory/immune changes observed in patients with Huntington's disease. Postmortem studies have shown increased microglia activation and astrocytosis in brain from patients who suffered from HD. In addition, increased expression of inflammatory mediators, such as cytokines and proteins related to intracellular pathways, was found in brains from patients with HD. Studies using PET also demonstrated increased microglial activation in HD. Cerebrospinal fluid samples from patients with HD presented increased levels of inflammatory mediators. In addition to changes in the central nervous system, patients with HD also display increased peripheral levels of inflammatory mediators, such as cytokines, chemokines, and complement factor proteins, besides alteration in monocyte activity and intracellular signaling proteins. A2M = alpha-2-macroglobulin; Akt = protein kinase B; APOA4 = apolipoprotein A4; CRP = C-reactive protein; HD = Huntington's disease; Ig = immunoglobulin; IL = interleukin; JAK/STAT = janus kinase/signal transducer and activator of transcription; MCP = monocyte chemoattractant protein; MIP = macrophage inflammatory protein; MMP = matrix metalloproteinase; NF-*κ*B = nuclear factor kappa; PET = positron emission tomography; PGLYRP2 = peptidoglycan recognition protein 2; sTNFR1 = soluble TNF receptor type I; TNF = tumor necrosis factor. ^*∗*1^One study found no change in circulating levels of TNF-*α* in HD. ^*∗*2^One study found that patients with HD presented higher serum levels of IgA than controls. ↑ = increase; ↓ = decrease; ↔ = no change.

A schematic summary of inflammatory/immune changes evidence from human studies is presented in Figure 1.

## 4. Anti-Inflammatory Drugs for the Treatment of HD

Based on data about immune/inflammatory changes in HD, some research groups have attempted to show the potential effects of immunomodulatory- and/or anti-inflammatory-based therapies in HD. The majority of studies are still preclinical; that is, they used* in vitro* cell cultures and animal models of HD for testing these strategies.

For instance, chronic treatment with the selective cyclooxygenase- (COX-) 2 inhibitors celecoxib and meloxicam attenuated behavioral and biochemical changes in a quinolinic acid-induced rat model of HD [48]. Another study, however, failed to demonstrate positive effects of the nonselective COX inhibitor acetylsalicylate or the selective COX-2 inhibitor rofecoxib in N171-82Q and R6/2 HD transgenic mice [49].

Minocycline and cannabinoids have also been tested in HD. Although not belonging to the classical definition of anti-inflammatory drugs, they are known to present anti-inflammatory properties. Minocycline is a second-generation tetracycline that has been in therapeutic use for over 30 years. In addition to its antibiotic properties, minocycline can exert a variety of biological actions, including anti-inflammatory and antiapoptotic activities [50]. A meta-analysis conclude that minocycline exerts neuroprotective effects in rodent models of neurodegenerative diseases, including HD. This effect is explained, at least in part, by minocycline anti-inflammatory, antiapoptotic, and antioxidant activities [51]. The evidence from preclinical studies in HD supported further testing in patients with HD. A clinical study (NCT00277355, Table 1, results published in [52]) showed that minocycline at 100 and 200 mg/day was well tolerated during 8-week treatment. The study involved 60 patients who were randomly assigned to receive placebo (*n* = 23), minocycline 100 mg/day (*n* = 18), or minocycline 200 mg/day (*n* = 19). However, no efficacy was observed, with no effect on the UHDRS scores. The short time period treatment might explain the lack of efficacy [52]. Corroborating these results, minocycline 200 mg/day was considered safe and well tolerated in a 6-month treatment protocol. Here again, no noticeable changes were reported in cognitive and motor symptoms as evaluated by the Mini-Mental State Examination, UHDRS, and Abnormal Involuntary Movement Scale [53].

Due to their anti-inflammatory properties, cannabinoids have been studied as a potential therapeutic approach in neuroinflammatory diseases [54]. A case report described a great improvement in chorea and behavioral symptoms, mainly irritability, with nabilone [55]; however, this was not confirmed by a clinical trial. A crossover study involving 44 patients with HD evaluated the benefits of nabilone over placebo on UHDRS measures and the Neuropsychiatry Inventory. Although nabilone was considered safe and well tolerated, there was no significant difference on the evaluated outcomes [55]. The use of cannabidiol was considered safe and well tolerated in a clinical trial conducted for 6 weeks with 15 patients with HD. Again, no significant improvement in clinical outcomes was observed [56]. Whereas these two clinical trials indicate lack of benefit, both were underpowered to detect differences, and thus no definite conclusions can be drawn [57].

Based on the evidence of increased levels of TNF-*α* in HD, one study investigated the therapeutic potential of XPro1595, a dominant negative inhibitor of soluble TNF-*α*. XPro1595 suppressed the inflammatory responses in two* in vitro* models: (i) primary astrocytes-enriched culture isolated from a transgenic mouse model (R6/2) exposed to LPS and (ii) human astrocytes-enriched culture derived from induced pluripotent stem cells (iPSCs) of patients with HD stimulated with cytokines. Moreover, XPro1595 protected the cytokine-induced toxicity in both* in vitro* models.* In vivo*, XPro1595 decreased TNF-*α* levels in the cortex and striatum, improved motor function, reduced caspase activation, diminished the amount of mutant HTT aggregates, increased neuronal density, and decreased gliosis in the brain of R6/2 mice [58].

Laquinimod is an immunomodulator that downregulates both proinflammatory cytokine production in peripheral blood mononuclear cells and astrocytic and microglial activation in the brain. Laquinimod was able to dampen hyperreactive production of cytokines from monocytes exposed to LPS harvested from both premanifest and symptomatic HD mutant human carriers [59]. A clinical trial is currently recruiting patients with HD in order to investigate the safety and efficacy of laquinimod (NCT02215616, Table 1).

Other drugs with potential anti-inflammatory/immunomodulatory effects have been tested in HD. The studies registered at *clinicaltrials.gov* are shown in Table 1. In addition to laquinimod, VX15/2503 is also currently recruiting participants (NCT02481674). VX15/2503 is a monoclonal antibody that blocks the activity of Semaphorin 4D, a protein known to contribute to neuroinflammation [60].

## 5. Conclusions

The contribution of neuroinflammation to neurodegeneration has previously been defined. However, the role of CNS and peripheral inflammatory changes in HD is still poorly understood. It is not clear whether inflammatory changes result from neurodegeneration and/or represent an independent pathological mechanism in HD. It is important to refine the understanding of the more specific immune/inflammatory mechanisms that are involved in HD. This will allow the development of more effective anti-inflammatory-based strategies. In addition, it remains to be investigated whether peripheral alterations mirror CNS changes and the putative routes of these interactions.

Although there is no evidence of peripheral immune cell infiltration into the brain of patients with HD, there are consistent results showing other neuroinflammatory processes in HD. First, postmortem and PET studies described microglial activation. Second, the levels of inflammatory mediators are increased in the brain and CSF of patients. Third, the levels of inflammatory mediators are also increased in peripheral blood from patients. Inflammation is likely an early event in the pathological process given that immune activation is shown to be present up to 15 years before symptom onset. It remains a matter of debate whether the inflammatory response is an active or a reactive (or both) mechanism in HD pathophysiology. Further studies are needed, mainly to help to study inflammation as a valid target for new therapeutic interventions to halt the progression of HD.

## Figures and Tables

**Table 1:** Clinical trials performed for testing anti-inflammatory-/immunomodulatory-based therapies in Huntington's disease.

Study	Drug	Trial design	Endpoint classification	Estimated enrollment	Outcomes	Status
NCT02481674	VX15/2503	Multicenter, randomized, double blind, placebo controlled, phase 2 trial.	Safety/efficacy	84	No published results.	Currently recruiting. Estimated primary completion date: August 2018.

NCT02215616	Laquinimod	Multicenter, randomized, double blind, placebo controlled, phase 2 trial.	Safety/efficacy	400	No published results.	Currently recruiting. Estimated primary completion date: August 2017.

NCT01502046	Cannabinoids: delta-9-tetrahydrocannabinol (THC) and cannabidiol (CBD)	Randomized, double blind, crossover, placebo controlled, phase 2 trial.	Safety	25	No published results.	Completed.

NCT00146211	Ethyl-EPA (Miraxion*™*, phospholipase A2 inhibitor)	Multicenter, randomized, double blind, placebo controlled, phase 3 trial.	Efficacy	300	^1^Ethyl-EPA was generally well tolerated. Ethyl-EPA was not beneficial in patients with Huntington's disease. At 6 months, the Total Motor Score 4 point change for patients receiving ethyl-EPA did not differ from that for those receiving placebo. No differences were found in measures of function, cognition, or global impression [61].	Completed.

NCT01357681	Epigallocatechin gallate (EGCG)	Multicenter, randomized, double blind, placebo controlled, phase 2 trial.	Efficacy	54	No published results.	Completed.

NCT00029874	Minocycline	Randomized, double blind, placebo controlled, phase 1/phase 2 trial.	Safety/efficacy	63	No published results.	Completed.

NCT00277355	Minocycline	Multicenter, randomized, double blind, placebo controlled, phase 2/phase 3 trial.	Safety/efficacy	114	^2^Minocycline at 100 and 200 mg/day for 8 weeks was well tolerated. No adverse events occurred more often with minocycline use [52].	Completed.

^1^Reference [61].

^2^Reference [52].
